# Buffalo Lithia Waters for Euræmia, Albuminuria of Pregnancy, Suppression of Urine in Yellow Fever, Menstrual Disorders, and Uric Acid Diathesis

**Published:** 1879-07-20

**Authors:** John W. Williamson

**Affiliations:** Tenn.


### *BUFFALO LITHIA WATERS FOR URÆMIA, ALBUMINURIA OF PREGNANCY, SUPPRESSION OF URINE IN YELLOW FEVER, MENSTRUAL DISORDERS, AND URIC ACID DIATHESIS.*

BY JOHN W. WILLIAMSON, M. D., OF TENN.

Being a native of, and until after the war a practitioner in, the Southside Section of Virginia, my attention has often been drawn to the virtues of Buffalo Lithia Spring No. 2. I have now prescribed this water for years, and it may henefit your readers to know my experience with it.

The first *test* that I ever made of it was in a case of uræmic poisoning occurring in a member of my own family. After a signal failure of every remedy that could be suggested by several eminent medical men, and when the condition of the patient was regarded as well nigh hopeless, a trial of this water was determined on. The result was relief from the threatening symptoms—so prompt and decided as to be almost incredible to any one but an eye-witness. If there be such a thing among medicinal agents as a specific, I think it may be fairly claimed for this water, that it is a *specific for urœmic poisoning.*

I read with much interest the remarks of Dr. James B. McCaw before the Richmond Academy of Medicine (reported in the December number, 1878, of your journal), concerning the Buffalo Waters, and especially the statement that he had found them of great value in the albuminuria of pregnant women. Both albuminuria and uræmic poison being fruitful of serious disturbance, and oftentimes of imminent danger to women during pregnancy, from what has been said, the use of these waters as a prophylactic during this period would seem to be very strongly indicated.

Their great value in malarial diseases and sequelæ has been most abundantly and satisfactorily tested; and I have no question that it would have been a valuable auxiliary in the treatment of the epidemic of yellow fever which so terribly afflicted the Mississippi Valley during the past summer. I prescribed it myself, and it gave prompt relief in a case of suppression of urine in yellow fever, and decidedly mitigated other distressing and dangerous symptoms. The patient recovered, but how far the water may have contributed to that result (having prescribed it in but a single case) I, of course, cannot undertake to say. There is no doubt, however, about the fact that its administration was attended by the most beneficial results.

While there is nothing in the analysis of the water that would indicate its special adaptation to such cases, I have found it of great potency and value in uterine diseases—particularly in the various disturbed conditions of the menstrual function.

Two cases of uric acid gravel have come under my observation in which this water gave perfect, and, I have reason to believe, permanent relief. As a nerve tonic, I think there is nothing known to the profession at all equal to it, and its *modus operandi* is, I take it for the most part, through the nervous system. Without going further into detail, I will add that this water is the most extensively applicable remedy in diseased conditions of the system, especially those involving morbid secretions, known to me.—*Virginia Medical Monthly.*

